# Aldosterone and the mineralocorticoid receptor in the cerebral circulation and stroke

**DOI:** 10.1186/2040-7378-4-21

**Published:** 2012-10-30

**Authors:** Quynh N Dinh, Thiruma V Arumugam, Morag J Young, Grant R Drummond, Christopher G Sobey, Sophocles Chrissobolis

**Affiliations:** 1 Department of Pharmacology, Monash University Wellington Road, Clayton, Victoria, 3800, Australia; 2 School of Biomedical Sciences, University of Queensland, Brisbane, Queensland, 4072, Australia; 3 Prince Henry’s Institute and the Departments of Physiology and Medicine, Monash University, Clayton, Victoria, 3168, Australia

**Keywords:** Aldosterone, Mineralocorticoid receptor, Stroke, Vascular remodelling

## Abstract

Ischemic stroke is a leading cause of morbidity and mortality worldwide. Elevated plasma aldosterone levels are an independent cardiovascular risk factor and are thought to contribute to hypertension, a major risk factor for stroke. Evidence from both experimental and human studies supports a role for aldosterone and/or the mineralocorticoid receptor (MR) in contributing to detrimental effects in the cerebral vasculature and to the incidence and outcome of ischemic stroke. This article reviews the evidence, including the protective effects of MR antagonism. Specifically, the effects of aldosterone and/or MR activation on cerebral vascular structure and on immune cells will be reviewed. The existing evidence suggests that aldosterone and the MR contribute to cerebral vascular pathology and to the incidence and outcome of stroke. We suggest that further research into the signaling mechanisms underlying the effects of aldosterone and MR activation in the brain and its vasculature, especially with regard to cell-specific actions, will provide important insight into causes and potential treatments for cerebrovascular disease and stroke.

## Background

Elevated plasma aldosterone level is an independent cardiovascular risk factor [1,2]. The mineralocorticoid receptor (MR) is known to be expressed in brain [3], blood vessels [4-6] and heart [7,8] as well as its classical site of expression in epithelial tissues such as the distal nephron. The MR is a member of the nuclear receptor superfamily and comprises an N-terminal domain, a central DNA-binding domain and a hinge region linked to a C-terminal ligand-binding domain. The MR has two physiological ligands, aldosterone and cortisol (corticosterone in rodents). It is established that in epithelial tissues aldosterone requires the enzyme 11β-hydroxysteroid dehydrogenase (11-βHSD2) to activate the MR, since 11-βHSD2 metabolises cortisol to cortisone [9]. Cortisol and corticosterone circulate at 100-1000 times the concentration of aldosterone, thus in the absence of 11-βHSD2 and under conditions of normal cortisol levels, the MR would be occupied by cortisol [10]. Co-localisation of 11-βHSD2 and the MR has been demonstrated in the vasculature (i.e. in endothelial and smooth muscle cells) [11-13], suggesting that aldosterone interacts with the MR in the vasculature.

Patients with primary aldosteronism (characterized by an overproduction of aldosterone) suffer stroke and cardiovascular events more frequently [14] than essential hypertensive patients despite having lower blood pressure, suggesting that elevated plasma aldosterone increases the risk of these events in a blood pressure-independent manner. Ischemic stroke is caused by interruption of blood flow to the brain, and deleterious stimuli which alter cerebral vascular structure and function will ultimately adversely influence cerebral blood flow [15]. Therefore, in humans with underlying cardiovascular risk factors, detrimental vascular actions of aldosterone, perhaps acting via the MR, may contribute to the pathophysiology of hypertension and stroke. The purpose of this article is to review evidence for a contributing role of aldosterone and the MR in stroke in human and experimental studies. Deleterious cerebral vascular actions of aldosterone and MR activation, including arterial remodeling, and recent evidence regarding effects on immune cells following ischemic stroke will be discussed.

## Aldosterone and the MR

Aldosterone, synthesized from cholesterol in the adrenal cortex, targets the distal nephron of the kidney to promote sodium and water retention, and potassium excretion, thus modulating electrolyte and fluid homeostasis and blood pressure [2]. Given its well known actions on the MR expressed in epithelial cells, aldosterone was traditionally thought to have an exclusive role in the kidney. However mounting evidence suggests that MR is also expressed in non-epithelial tissues, including the brain, vasculature, cardiomyocytes and immune cells such as macrophages [16]. Indeed, both aldosterone production and MR expression have been detected in the brain [3], blood vessels [4-6] and heart [7,8].

**Figure 1:**
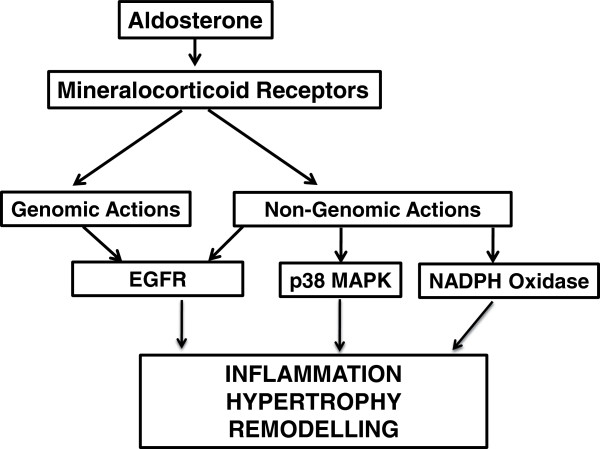
**Schematic diagram illustrating examples of genomic and non-genomic pathways contributing to vascular remodelling following mineralocorticoid receptor activation by aldosterone.** Figure based on text and ref [39].

The signalling actions of aldosterone may be either genomic or non-genomic (Figure 1). Genomic actions reflect the classic model of aldosterone action and involve it binding to the MR in the cytoplasm, resulting in MR release from chaperone proteins, dimerization of the receptor and translocation to the nucleus where it binds to hormone response elements on promoters leading to activation of gene transcription [17]. By contrast, rapid, non-genomic actions of aldosterone occur when it binds to MR or other receptors on the cell surface [17] (e.g. G protein coupled receptor 30 [GPR30], and possibly the angiotensin II type 1 receptor [AT_1_R]) [18,19] to activate second messenger systems. 

## Effects of MR antagonism on stroke outcome

MR antagonists appear to have beneficial effects in experimental models of stroke. Spironolactone and the more MR-selective compound, eplerenone [20] can both markedly prevent stroke from occurring in stroke-prone spontaneously hypertensive rats (SHRSP) maintained on a 1% NaCl/stroke-prone diet [21,22]. In both studies, control SHRSP showed signs of stroke and died by 16 [21] or 18 [22] weeks of age, whereas all SHRSP treated with spironolactone, and >75% treated with eplerenone, appeared normal and survived until 19 weeks of age before they were culled. Severe cerebrovascular and parenchymal lesions in brains of SHRSP were also reduced in both spironolactone- and eplerenone-treated animals [21,22], suggesting that MR inhibition protects against stroke incidence through actions on both vascular and parenchymal tissues. Additionally, MR antagonism appears to improve outcome after experimental ischemic stroke. Specifically, pretreatment with either spironolactone [23] or eplerenone [24] reportedly reduces infarct size following middle cerebral artery occlusion in SHRSP [23] and in mice [24].

The aforementioned studies provide strong evidence for a role of MR in increasing the incidence and adverse outcomes in rodent models of stroke, although the cellular location(s) of the MR involved was unknown. However, a recent and important study by Frieler and colleagues [25] has provided evidence that MR expressed on myeloid cells (i.e. non-lymphocytic leukocytes) may play an important role in post-stroke outcome. Following cerebral ischemia, a smaller infarct volume was produced in myeloid cell MR-deficient mice compared with control mice, consistent with MR-expressing circulating immune cells mediating post-ischemic neuronal injury [25]. Interestingly, the same group also provided evidence that myeloid cell MR expression regulates macrophage polarization [26], in that deletion of myeloid MR results in an M2 (or ‘alternate’, as opposed to M1 or ‘classical/pro-inflammatory’) macrophage polarization [26].

Macrophage response profile appears to occur according to the polarization state and thus depends on two main groups of T lymphocyte-derived cytokines. For example, Th1-type cytokines (e.g. IFNγ) induce M1 macrophages to display classical pro-inflammatory responses needed for killing parasites, whereas Th2-type cytokines (e.g. interleukins 4, 5, 13), which induce the M2 macrophage phenotype, are also important in the immune response but tend to instead exert an anti-inflammatory action [27]. In addition to myeloid MR activation promoting a proinflammatory M1 state, aldosterone also stimulates several M1 mediators to be generated by peritoneal thioglycolate–elicited macrophages (e.g. TNFα, RANTES, IL-12) with the observation that TNFα expression may be inhibited by eplerenone indicating that the pro-inflammatory action of aldosterone was MR-mediated [26]. In addition, a number of M1 markers (IL-1β, TNFα, IL-6, MCP1, MIP1α) were elevated in the ischemic brain hemisphere of control mice, but these changes were suppressed in myeloid MR-deficient mice. Similarly, there was a marked increase in immunoreactive Iba1^+^ cells in the ischemic core of control mice (indicative of activated microglia and macrophages), which was significantly weaker in myeloid MR-deficient mice [25]. Together, these findings suggest that MR activity may play an important role in regulating immune cell function during the inflammatory response following cerebral ischemia, thus worsening stroke outcome.

## Role of aldosterone in stroke outcome

### Animal studies

Whilst it is likely that much of the evidence reviewed above implicating roles for MR activity in stroke indirectly reflects actions of its main endogenous agonist, aldosterone, there is nevertheless additional evidence that more directly implicates a role for this mineralocorticoid in stroke outcome. Treatment with deoxycorticosterone acetate (DOCA), often in combination with uninephrectomy and salt treatment, is commonly used to model pathology caused by aldosterone excess. However, even administration of DOCA alone to normal rats (i.e. in the absence of uninephrectomy and salt treatment) can result in an increased cerebral infarct size following permanent middle cerebral artery (MCA) occlusion, an effect that was associated with increases in both vessel wall thickness and wall: lumen ratio, and decreased lumen and outer diameters of the MCA [28]. Thus, increased levels of mineralocorticoids may contribute to stroke outcome, and such an effect may at least partially result from effects on cerebral blood vessel structure.

Further evidence for a role of aldosterone in outcome after stroke comes from studies of standard anti-hypertensive therapies. Angiotensin converting enzyme inhibitors (ACEIs) and angiotensin II (Ang II) receptor blockers (ARBs) are frontline therapy for hypertension treatment [29], however their beneficial effects in stroke may include the suppression of aldosterone levels and be partly blood pressure-independent [30]. For example, in SHRSP maintained on a high salt diet, the ACEI captopril [31-33] and the AT_1_R anatagonist losartan [33] increase post-stroke survival [31,32] and restore cerebral blood flow autoregulation [33], yet neither drug exerted an anti-hypertensive effect. In contrast, treatment with the diuretic, hydralazine, significantly lowered blood pressure but was inferior to captopril in improving post-stroke survival [31,32], consistent with the protection by ACEIs and ARBs being blood pressure-independent. In these studies, SHRSP treated with either captopril or losartan had lower plasma aldosterone levels than control SHRSP. Furthermore, when plasma aldosterone levels were restored to normal in captopril-treated SHRSP by infusion from implanted minipumps, the beneficial post-stroke effects of captopril were lost [31,32], as would be expected if the protection by captopril involved suppression of plasma aldosterone levels. Interestingly, although yet to be directly assessed in cerebral vessels, mesenteric arteries from SHRSP are reported to contain significantly higher aldosterone levels at an early age when compared to control rats [34].

Despite experimental evidence that short-term benefits of combination ARB and ACEI therapy may be due to suppression of elevated aldosterone levels, findings from clinical trials using ARBs and ACEIs indicate that, in some patients on combined ARB/ACEI therapy, plasma aldosterone levels can increase over the long term [35]. Thus it is likely that this phenomenon, termed ‘aldosterone breakthrough’, will have important clinical consequences given the non-epithelial actions of aldosterone in organs such as the brain, vasculature, heart and immune system, and may therefore need to be addressed perhaps by additional MR antagonism.

### Human studies

Clinical evidence appears to be consistent with data from experimental models indicating an important role for elevated aldosterone levels in stroke outcome [36], perhaps due to elevated aldosterone levels even in the absence of hypertension. For example, the incidence of cerebrovascular events (e.g. stroke, aneurysm, subarachnoid hemorrhage) in patients with glucocorticoid remediable aldosteronism is associated with elevated aldosterone levels and suppressed renin activity [37]. Similarly, a large retrospective study found an association between high aldosterone levels and the risk of stroke and transient ischemic attack that was independent of blood pressure and other risk factors [38]. Furthermore, patients with primary aldosteronism suffer more strokes than patients with essential hypertension, despite having lower blood pressure [39], and they have much higher rates of stroke than age-, sex-, and blood pressure-matched essential hypertensives [14]. Indeed, in recently published clinical studies, relative aldosterone excess (i.e. an increased aldosterone-to-renin ratio) has been identified as a predictor of stroke/transient ischemic attack, during both normal and high sodium intake [40].

## Gender effects on aldosterone levels and MR antagonism in stroke

### Human studies

Postmenopausal hypertension is largely dependent on mineralocorticoid receptor activation and is selectively sensitive to mineralocorticoid receptor antagonists [41]. Furthermore, in premenopausal women, plasma aldosterone levels are lower than in men [42], but after menopause there is no gender difference in plasma aldosterone levels [43,44].

### Experimental studies

Spironolactone and eplerenone were without effect on infarct size in female SHRSP despite a higher relative expression of MR in cerebral arteries in females than males [45], highlighting a potential sex difference in the utility of MR antagonists for stroke therapy. Another study using Wistar rats found that females were less sensitive to central MR antagonism (using the MR antagonist RU28318) than males [46]. Thus, in contrast to findings in human studies where MR appears to be important in post-menopausal women, in experimental studies females appear to be less sensitive to MR antagonism.

## Complex roles of aldosterone and the MR in the brain

Despite the evidence that aldosterone contributes to the incidence and outcome of stroke, and that MR inhibition may be protective under these pathological conditions, the MR is nevertheless likely to be important in the normal physiology of the brain. The receptor is most highly expressed in the hippocampus where it is plays a role in behavioral, cognitive and neuroendocrine regulation [47], and pharmacological evidence suggests it is neuroprotective in that it can play a role in neuronal cell survival after cerebral ischemia [48]. Indeed, as cerebral ischemia is known to increase MR expression in the hippocampus of humans [49], rats [48] and gerbils [50], this aspect of MR signaling could be beneficial for promoting neuronal survival. Furthermore, there is evidence that in mice with forebrain overexpression of the MR, neuronal death following transient global cerebral ischemia is reduced compared to controls [47].

One mechanism whereby the MR could reduce neuronal cell death is through reversing the pro-apoptotic effect of the glucocorticoid receptor (GR). Activation of the GR increases neuronal cell death in a hippocampal culture system, and this effect was significantly reduced in the presence of aldosterone. Although it was not examined whether that effect was MR-dependent, the addition of either spironolactone or RU28318 was found to increase neuronal cell death [51]. Thus, the effects of MR (and GR) on neuronal survival could occur via influencing the expression of regulatory proteins associated with apoptosis, and their overall effect (i.e. protective or detrimental) may be the opposite in hippocampus and cortex – the two locations in the brain where MR are normally expressed. For example, in rat hippocampus, GR activation increases the ratio of the pro-apoptotic protein, Bax, relative to the anti-apoptotic proteins, Bcl-2 and Bcl-x_L_, resulting in increased neuronal cell death, whereas in that study the opposite effect was found after MR activation [52]. However, MR expression occurred after cerebral ischemia in the striatum, and spironolactone treatment increased expression of neuroprotective factors and the number of migrating neuroblasts in that region [53].

## Aldosterone, MR, and cerebral artery remodelling

Blood vessels, including cerebral arteries, can undergo hypertrophy (i.e. increased cross-sectional area) and/or exhibit remodeling (inward or outward changes in diameter) in response to hypertension and other chronic stimuli [54]. Several types of cellular processes and signaling mechanisms may be associated with vascular remodeling, including fibrosis, rearrangement of vascular smooth muscle cells, or increased responsiveness to epidermal growth factor (EGF). EGF binds to the EGF receptor (EGFR) to activate cell growth and proliferation [23]. Inward remodeling in the cerebral vasculature can lead to a reduced lumen diameter and a less flexible vessel wall, which consequently impairs the ability of the cerebral vessel to dilate in response to ischemia [23].

Aldosterone, which is also reported to cause vascular remodelling by increasing media thickness [55], increases extracellular matrix proteins such as fibronectin in carotid arteries, an effect that is inhibited by eplerenone [56], suggesting cerebral vascular remodeling in response to elevated aldosterone is MR-dependent. MR located on vascular smooth muscle cells (VSMC) mediates contraction of the mesenteric artery in response to Ang II, and increased contraction in response to KCl and U46619 in aged mice was also dependent on MR in VSMC. Although MR expression in VSMC had no effect on lumen diameter or wall-to-lumen ratio, mesenteric arteries from mice lacking VSMC MR developed less spontaneous myogenic tone compared to controls [57]. These findings indicate an important role for VSMC MR in vascular reactivity. Increased expression of EGFR mRNA has been reported in rat cerebral vessels from SHRSP compared to control rats [23]. Spironolactone treatment resulted in increased lumen and outer diameters of the MCAs in SHRSP, as well as a reduced wall/lumen ratio without lowering blood pressure [58,59], suggesting that spironolactone alters cerebral vascular structure without a change in blood pressure.

## Immune cell MR as mediators of stroke damage

In addition to the concept that MR activity regulates infiltrating myeloid cell function during the inflammatory response following cerebral ischemia [25], there is further evidence for an effect of aldosterone on immune cells [60-62]}. Aldosterone stimulated leukocyte adhesion to human coronary artery endothelial cells that was abolished by spironolactone [61]. Immune cell (ie. monocytes/macrophages, as demonstrated by monocyte/macrophage specific antigen MOMA-2 immunostaining) infiltration in aorta in response to aldosterone was also prevented by Treg adoptive transfer [62]. Aldosterone augmented both CD8^+^ T cell activation by dendritic cells (which express MR), as well as IL-17 (pro-inflammatory cytokine) release by CD4^+^ T cells – effects that were inhibited by spironolactone and eplerenone [60]. These results were obtained from the use of bone marrow-derived dendritic cells, nevertheless they support the very new concept that aldosterone, perhaps acting via MR, can modulate dendritic cell function and promote T cell activation. Indeed, in association with their smaller cerebral infarct volume (discussed above), myeloid cell MR-deficient mice also have fewer activated microglia, macrophages and pro-inflammatory mediators (IL-1β and TNFα) in the ischemic core compared to control mice [25], implicating immune cell-expressing MR as important mediators of inflammation in ischemic stroke.

## Conclusions and future directions

**Figure 2:**
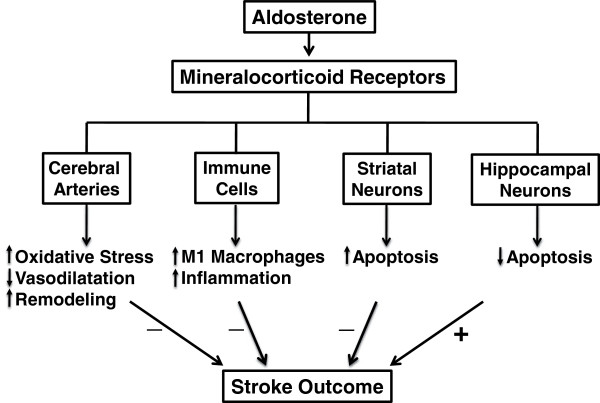
**Schematic diagram illustrating the potential cellular targets and ultimate effects of mineralocorticoid receptor activation by aldosterone on stroke outcome.** ‘↑’ = increased; ‘↓’ = decreased; ‘+’ = improved; ‘−’ = worsened.

It is now established in experimental stroke studies that activation of the MR contributes to a worse outcome following stroke, as well as altered structure of cerebral blood vessels. Clinical findings also indicate that elevated plasma levels of aldosterone may be a predictor of increased stroke risk (Figure 2). Nevertheless, although these data provide useful insight for the future development of novel stroke therapies, it is noteworthy that both aldosterone, and particularly the MR, may actually exert protective actions in certain brain regions following stroke (Figure 2). Therefore, in order to develop safe and effective therapies targeting these pathways, further research is needed to clarify the cellular and molecular mechanisms underlying the effects of aldosterone and MR activation in the brain and vasculature under physiological conditions as well as during cerebrovascular disease and stroke. The use of cell-specific MR-deficient mice in future studies will thus be crucial to this area of research. There is also a need to clarify if the clinical utility of MR antagonists is likely to be sex-specific.

## Abbreviations

MR: Mineralocorticoid receptor; 11β-HSD: 11β-hydroxysteroid dehydrogenase; GPR30: G-protein coupled receptor 30; AT_1_R: Angiotensin II type 1 receptor; SHRSP: Stroke prone spontaneously hypertensive rats; DOCA: Deoxycorticosterone acetate; MCA: Middle cerebral artery; ACEIs: Angiotensin converting enzyme inhibitors; Ang II: Angiotensin II; ARBs: Ang II receptor blockers; GR: Glucocorticoid receptor; EGF: Epidermal growth factor; EGFR: EGF receptor.

## Competing interests

The authors declare that they have no competing interests.

## Authors’ contributions

QND wrote the first draft and revised the manuscript. TVA, MJY and GRD revised the manuscript. CGS and SC drafted and revised the manuscript. All authors read and approved the final manuscript.
